# Mr. Wharton Jones on the Anatomy, Physiology, and Pathology of the Blood

**Published:** 1842-10

**Authors:** T. Wharton Jones

**Affiliations:** Lecturer on Anatomy, Physiology, and Pathology at the Charing-cross Hospital Medical School


### PART FOURTH.

### Medical Intelligence.

OBSERVATIONS ON SOME POINTS IN THE

### ANATOMY, PHYSIOLOGY, AND PATHOLOGY OF THE BLOOD.

By T. Wharton Jones, F.R.S.

Lecturer on Anatomy, Physiology, and Pathology at the Charing-cross Hospital Medical School.

The blood, as it circulates in the vessels of the living body, consists of a transparent colourless liquid, called *liquor sanguinis* or *plasma*, containing in suspension microscopical corpuscles of two kinds: the one red and in very great number, the other colourless and very few in number.

Liquor Sanguinis. The liquor sanguinis is scarcely an object of microscopical examination, except in connexion with the corpuscles suspended in it. The liquor sanguinis of frog's blood may be separated from the red corpuscles by filtration, but that of human blood cannot be obtained free from red corpuscles, except when the blood is drawn during inflammation, and certain other states of the system. In this case the liquor sanguinis, mixed, however, with colourless corpuscles, rises to the top in considerable quantity, and may, before coagulation takes place, be removed for examination. I shall return to this point, more particularly afterwards, when considering the formation of the buffy coat.

Red Corpuscles of the Blood. *Form.* The red corpuscles of the blood, though they have been called globules, are not in their ordinary state globular, but have the form, as is now generally known and admitted, of biconcave lenses, with the peripheral edge obtusely rounded; the biconcave lenticular form is proclaimed by the appearance which the corpuscle presents under the microscope, viz. the circumference as a broad bright ring and the centre as a dark spot; or, on the contrary, the circumference dark and the centre bright, according as one or other is in the focus of the microscope.

*Size.* In healthy blood, newly drawn, and unaltered by any reagent, the corpuscles are between $\frac{1}{3000}$ and $\frac{1}{3500}$ of an English inch broad, and about one fourth of that thick at the circumference, but thinner, of course, at the centre.

*Structure.* The red corpuscle is a vesicle, or cell, with thick walls, but in a collapsed flattened state. Its form is exactly such as a thick-walled vesicle, or cell, in a collapsed flattened state would present. The bright or dark ring represented by the circumference, and the dark or bright spot by the centre, according as one or other is in the focus of the microscope, are appearances which tally with those known to be presented by a collapsed thick-walled cell. Certain reagents by giving rise to exosmose, render the corpuscle flatter, and consequently bring more distinctly into view its lumen, and the double contour of its thick wall.

Misinterpreting the appearance presented by the double contour of the thick wall of the corpuscle, Dr. Martin Barry has described it as being produced by an annular fibre contained in the interior of the corpuscle. In his papers on the blood, he described the corpuscles as containing in their interior certain minute bodies, which he calls "*discs*," in shape flat, elliptical or circular, usually concave in the middle of the flat surface. He now finds that these "*discs*," by uniting, form "fibre." In mammalia, including man, says he, the "fibre," or filament, is simply annular, (hence, he says, the biconcave form of the corpuscle in this class,) but in the other vertebrata the "discs" contained within the corpuscle are too numerous for such a *ring*, therefore their arrangement forms a *coil*.

In regard to these statements it is to be observed: 1st, That the corpuscles which Dr. M. Barry delineates and describes as blood-corpuscles containing "*discs*" are not real red corpuscles, but the colourless ones known by the name of lymph-corpuscles; 2dly, That in the real red corpuscles of human blood there are certainly no "*discs*" to be seen; and 3dly, That the appearance which the real red corpuscle may be made to present by reagents, and which Dr. M. Barry interprets as an annular fibre in its interior, is simply the bright annular appearance above spoken of as being produced by the folding of the thick wall of the corpuscles; the double contour being rendered well defined by the great flattening which takes place. The figures of the red corpuscles of man given by Dr. Barry to show the alleged contained fibre are not true to nature, but appear to be exaggerations of that state of the corpuscle produced by reagents in which, at the same time that it is much flattened, its edge is beaded or as if bent here and there in opposite directions like some kinds of biscuits. The form, often assumed by the blood discs of the newt, of flask-like vesicles, with the appearance of a minute body protruding from their neck, and which Dr. Barry describes as the extremity of the filament in question, I have observed presented even by the human blood-corpuscle, but it was easy to see that the alleged fibre was nothing more than the substance of the corpuscle, changed in consistence by the reagent, drawn out, as a viscid matter is, into a thread.

The fibres which the fibrin may be observed to form in solidifying, are described by Dr. Martin Barry as fibres escaped from the interior of the corpuscles. He even affirms that he has noticed "the ring formed in the blood-corpuscle of man, and the coil formed in that of birds and reptiles, unwinding themselves into the straight and often parallel filaments of the coagulum; "changes which," he says, "may be also seen taking place in blood placed under the microscope before its coagulation."

The thick wall of the red corpuscle consists of two layers. The outer is transparent, colourless, structureless, and resisting, and constitutes about one half of the whole thickness of the wall. The inner layer is softer, and less resisting; and is that which is the seat of the colouring matter. The outer layer may be compared to the vitellary membrane of an ovum, the inner layer to the representative of the yelk in the mammiferous ovum.

By the addition of water, &c., to the blood, endosmosis takes place through the walls of the red corpuscles, which thus acquire the spherical form of a distended vesicle or cell, becoming at the same time so transparent as to be with difficulty perceived. Red corpuscles thus changed may be brought back more or less nearly to their original form by the reagents which give rise to exosmosis. Solution of iodine, besides doing this, tinges the wall of the corpuscle yellow, and thus renders it very distinct.

Water, acetic acid, &c., readily extract the colouring matter with which the inner layer of the corpuscle is impregnated. The distention of the corpuscle, which at the same time takes place, if considerable, causes the bleached inner layer to be broken up, and separated into minute colourless granules, or into streaks, while the outer layer remains, though collapsed. In examining the corpuscles of frog's blood under the microscope, I have sometimes observed that when dilute acetic acid was added, the inner layer suddenly gave way with a jerk.

The red corpuscle of the blood of the frog presents out of the body a very distinct nucleus, but not, it is said, when the blood is observed circulating in the web of the living animal's foot; hence, it has been suggested that the nucleus may be formed only after abstraction of the blood from the body. The cause, however, of the nucleus not being always distinctly seen in the blood while circulating, appears to be that the corpuscles are then somewhat more distended than they are when out of the body.

Does the *red* corpuscle of human blood possess a nucleus? This is a question which has been variously answered. Some have spoken familiarly of a nucleus, some doubtingly, and some have altogether denied the existence of one. In the unaltered red corpuscles there is no appearance of nuclei;* but when to the blood some reagent has been added, for instance, acetic acid, minute shining par-

* Of course the central depression is not to be confounded with a nucleus.

ticles, about one fourth or one fifth of the diameter of the corpuscles, come into view, but not in connexion with the corpuscles. These minute particles float about quite free, and exhibit molecular movements. They are nothing but particles of fibrin, or albumen, precipitated by the reagent, as may be proved by adding to liquor sanguinis, or even serum, in which there is not a single red corpuscle or particle to be seen, some reagent, as acetic acid, when the minute particles under notice will be produced in great quantity. When liquor sanguinis is used, some larger particles are also produced.

This question of a nucleus has no reference whatever to the colourless corpuscles which exist in small number in the blood dispersed among the red corpuscles, for they are well known to present one or more nuclei in their interior after being acted on by acetic acid. In his second paper on the corpuscles of the blood, Dr. Martin Barry speaks of the nucleus of the red corpuscles being composed of several parts instead of being one object, as is, according to him, usually considered to be the case. What he delineates, however, as blood-corpuscles of man, after the addition of acetic acid, most certainly are not the red corpuscles which constitute the mass of the blood, but the perfectly distinct colourless ones just referred to, and to be noticed more particularly below.

The red corpuscles, it is well known, are extremely prone to become granulated on the surface, especially at the circumference, which thus appears beaded and notched. Not unfrequently one bead is observed about the centre, which might be put down as a nucleus, but it is not so. The granulated appearance seems to be owing to a contraction of the inner, and a wrinkling of the outer, of the two layers of the wall of the corpuscle. The circumstance that the corpuscles so changed are less in diameter than natural, besides other appearances, support this view. The granulated or mulberry appearance may be at once produced by pressure, e. g. by pressing down closely the superjacent thin plate of glass on the minute quantity of blood under examination. It may also be readily produced by certain reagents, as, for instance, a solution of common salt, oil, &c. In our examinations of the blood some granulated corpuscles are generally seen towards one or other side of the field ; this I believe, is owing to the evaporation of the fluid part of the blood at the edges of the superposed plate of glass, allowing the latter to be more closely pressed down on the blood.

It appears to be the change to that state giving rise to the mulberry appearance just noticed that Dr. Martin Barry describes as "progressive division of the blood-discs into globules." He considers it to be a vital process, and that the globules thus alleged to be produced are the foundations of new corpuscles of the blood. It has been already shown that Dr. Martin Barry mistakes the colourless for the red corpuscles of blood; we now find that he mistakes decomposed and distorted red corpuscles of blood for living blood-corpuscles, undergoing progressive division. Under this erroneous impression he describes the granulated or mulberry state of the red blood-corpuscles, as an advanced degree of that which the colourless or lymph-corpuscles of the blood present, after being acted on by acetic acid.

The red corpuscles are yielding and elastic, so that they readily change shape when slightly pressed upon, like partially filled bladders, which, indeed, they are, and as readily regain their original form when they have escaped from the compressing agent. In consequence of this property the corpuscles glide along in their vessels, with great ease accommodating themselves to all obstacles and to each other. In a mixture of blood and pus the red corpuscles are observed to yield in the most extraordinary manner, so as to accommodate themselves to obstacles. Thus, in order to pass through a narrow channel, they will be drawn into a mere filament, and yet, when free, immediately regain their original form. Their capability of being moulded into various shapes depends on the state of distention of the red corpuscles, and this again on the nature of the liquid in which they are suspended.

Dr. Barry describes and delineates corpuscles found in fluid having nearly the colour of blood taken from an abscess. The fluid examined by Dr. B. was evidently no other than a mixture of pus and blood, and he has mistaken the changes which the red corpuscles of blood undergo in consequence of the reagency of pus, for transformation of the blood-corpuscle into a pus-globule.

Mr. Gulliver describes very well these changes of the red corpuscles which Dr. Barry has so completely misinterpreted.*

The rapid and incessant changes in form of the altered blood-corpuscles which Dr. Barry speaks of, can have been owing merely to his having ill observed the turning over and over from side to edge, and from edge to side, of the irregularly-shaped corpuscles. In reference to the same point, Dr. Nasse says—"I do not exactly know what Barry means, but probably it is merely an appearance produced by imbibition."

Colourless Corpuscles of the Blood. The colourless corpuscles are slightly larger than the red corpuscles, appear finely granulated on the surface, and strongly refract light. They are specifically lighter than the red corpuscles; hence, when a minute drop of blood is mixed with a similar drop of water, acetic acid, &c., they float above, and are, therefore, seen in a different focus from the red corpuscles; hence, also, they are found suspended in the liquor sanguinis, which rises to the top, when a buffy coat is to be formed. Though comparatively few in number, the colourless corpuscles in the blood are sufficiently numerous for two or three to be observed at once in a minute drop of pure blood thinly spread out. In a drop of blood mixed with water they are perhaps more readily detected at once, and in greater number from their being in a different focus from the red corpuscles, and from their not being, like the latter, rendered indistinct by the action of the water.

*Structure.* By the action of acetic acid several nucleus-like bodies connected together become visible, and the delicate membrane composing the external wall of the corpuscle is very much distended, so that the diameter of the corpuscle is now about one third or more greater than that of the red corpuscle.

Coagulation of the Blood. In the course of a few minutes after its escape from the body the blood coagulates into a soft red mass, like jelly. By the contraction of this mass, which slowly ensues, a yellowish liquor is gradually squeezed out. The solid and liquid matters into which the blood is thus resolved are known by the names of *crassamentum* and *serum.*

*Intimate part of the process of coagulation.* The crassamentum and serum into which the blood is resolved by coagulation, it is to be noted, are not the same, respectively, as the red corpuscles and liquor sanguinis, above mentioned, as the components of the blood, in respect of form, while it is still circulating in the body, and for a short time after it has been drawn.

The annexed table† illustrates the differences between the components, in respect of form, of living, and of coagulated blood.

| Living Blood. | | *Chemical Components.* | | Coagulated Blood. |
|---|---|---|---|---|
| Living Blood. | *Liquor Sanguinis.* | Water | *Serum.* | Coagulated Blood. |
| | | Various salts | | |
| | | Fatty matters | | |
| | | Extractive do. | | |
| | | Albumen | | |
| | | Fibrin. | *Crassamentum.* | |
| | *Red Corpuscles.* | | | |

Coagulation of the blood is due to solidification of the fibrin, which was previously in a fluid state in the liquor sanguinis. The red corpuscles have no posi-

* "The corpuscles are sometimes rather humid on the surface, lenticular, and occasionally cup-shaped. They are often swollen at the edges, which, in consequence, project towards the centre, thus producing there triangular, oval, or irregular depressions. The cup-shaped variety is rather frequent in corpuscles which have been mixed a little while with saline solutions, and it is not uncommon in man, particularly among the particles of purulent or other morbid fluids."—(Appendix to translation of Gerber's Anatomy.)

† Similar Tables have already been given by Mandl and Bruns.

tive share in the process, and though contained in the crassamentum, they form no necessary part of it. The crassamentum is formed essentially of the solidified fibrin.

The simplest demonstration of the proposition just stated is the well-known process of stirring newly-drawn blood, when the fibrin, as it solidifies, is precipitated on the rod in the form of a soft elastic fibrous substance, whilst the red corpuscles remain perfect and entire, mixed with the serum. A more satisfactory, though not so simple a way consists in separating, by artificial means, the liquor sanguinis, still containing the fibrin in solution, from the red corpuscles. The liquor sanguinis, thus separated, may then be observed to coagulate, and afterwards to resolve itself into a colourless crassamentum and serum.

Hewson's method of separating the liquor sanguinis from the red corpuscles consisted in preventing blood from coagulating by dissolving in it, as it flows from the vein, sulphate of soda. "The red particles," Hewson* remarks, "readily subside, and the surface of the mixture becomes clear and colourless; and being poured off from the red part, it is found to contain the coagulable lymph, which can be coagulated, and thus separated, by the addition of water." Various other neutral salts, besides the sulphate of soda, possess the property of keeping the blood fluid, and yet allow it afterwards to jelly on being mixed with water. Some salts again, though they keep the blood fluid, do not allow it to jelly when mixed with water. The most elegant and convincing method is that pointed out by Professor Müller. It consists in receiving frog's blood on a filter, which, in consequence of the size of the red corpuscles, may be porous enough to allow the liquor sanguinis to pass quickly through. The pure liquor sanguinis thus obtained soon coagulates, and by and by resolves itself into colourless crassamentum and serum. It is to be remarked that for this process to succeed, the filtering paper must neither be so loose in texture as to give passage to the corpuscles; nor so close as to retard the oozing through of the liquor sanguinis, and thus give time for coagulation to take place.

The question has been much agitated whether coagulation of the blood be owing to purely physical causes, or whether it is a vital process excited by an external stimulus, or whether it is the effect of loss of vitality. Instead of attempting to determine the validity of one or other of these opinions it may be asked with Henle,† why does the blood circulating in the vessels *not* coagulate? A satisfactory answer, perhaps, to this question may be given by saying, that the liquor sanguinis being constantly pervaded by the red corpuscles is elaborated by them, and that the coagulable part of the blood is taken up as quickly as it is formed. If the view here taken be well founded, it may serve as a step towards the solution of the above questions.

MODE OF FORMATION OF THE BUFFY COAT. In the healthy condition of the blood, no separation of the liquor sanguinis from the red corpuscles takes place naturally; but in certain states of the system, in inflammations especially, the blood soon after being drawn undergoes the separation to a greater or less amount. The liquor sanguinis separated from the red corpuscles collects at the top, and its fibrin in a short time coagulating, the well-known buffy coat is formed. In such a case, if, before coagulation, some of the clear liquor sanguinis, as soon as it rises to the top in sufficient quantity, be removed with a spoon, it will in a short time be found to coagulate, and to separate into a colourless crassamentum and serum.

In the liquor sanguinis which rises to the top in inflammatory blood, colourless corpuscles are found in great number. The cause of the colourless corpuscles rising to the top with the liquor sanguinis is, I believe, their small specific gravity, an attraction for the liquor sanguinis, and a want of attraction for the red corpuscles, to be noticed below. Their great number appears to be owing to this, that the whole of the corpuscles of this kind, which were diffused through the quantity of blood drawn, are now collected in the small quantity of liquor sanguinis

* Experimental Inquiries: Part the first, containing an Inquiry into the Properties of the Blood, &c. 2d ed. p. 12. London, 1774.

† Allgemeine Anatomie, &c., 1841.

which has risen to the top. I do not deny but that the colourless corpuscles may be more numerous in buffy than in healthy blood, but the explanation just given renders it probable that they are actually not so very much more so as might at first have been supposed. In corroboration of this I would add, that in all my microscopical examinations of blood, which afterwards became buffed, I cannot say that a very great increase in the number of colourless corpuscles was noticed.

When the fibrin of the liquor sanguinis coagulates to form the buffy coat, the colourless corpuscles are entangled among the fibres and minute granules into which the fibrin solidifies. A portion of the buffy coat, examined under the microscope, thus appears as a fibrous tissue, containing, interspersed through it, nucleated corpuscles.

The colourless corpuscles contained in the liquor sanguinis which has risen to the top in inflammatory blood, have been supposed to form by their coalescence the buffy coat. This inaccurate interpretation of the matter, first given by Mr. Addison,* has been assented to by Dr. Barry.

The separation of the liquor sanguinis, to a greater or less amount, from the red corpuscles in inflammatory blood, and its non-separation in healthy blood, has been variously accounted for. A very generally received view is, that the separation of the liquor sanguinis from the red corpuscles giving rise to the buffy coat, is owing to the blood, on such occasions, coagulating more slowly, and the corpuscles thus having time to subside. In regard to this, however, it is sufficient only to remark, that the separation of the liquor sanguinis from the red corpuscles may often be observed to have taken place long before the time at which healthy blood usually coagulates.

As will be seen in the course of this paper, I do not deny that the greater specific gravity of the red corpuscles has some share in their separation from the liquor sanguinis, but that it plays a subordinate part merely in the process, is proved by the circumstance that "the separation of the fibrin (liquor sanguinis) from the colouring matter (red corpuscles) in such cases, takes place in films of blood so thin as not to admit of a stratum of the one being laid above the other; they separate from each other laterally, and the films acquire a speckled or mottled appearance, equally characteristic of the state of the blood as the buffy coat itself, as shown by Schroeder Van der Kolk." †

The separation of the liquor sanguinis from the red corpuscles, which occurs in healthy blood kept fluid by a neutral salt, and which appears to be owing really to subsidence of the red corpuscles, is no illustration of the way in which the separation takes place in inflammatory blood; as in the former case, the separation proceeds much more slowly than in the latter. By the action of the neutral salt the red corpuscles are contracted and rendered specifically heavier; but it is to be remembered that the liquor sanguinis will at the same time be rendered specifically heavier also, in consequence of the salt dissolved in it. That the specific gravity of serum, at least, is increased in greater proportion than that of the red corpuscles, by admixture with a neutral salt, seems to be proved by the experiment of taking two portions of a mixture of serum and red corpuscles, and adding sulphate of soda to the one and none to the other. In that to which no salt has been added, the corpuscles subside both more quickly and more completely. It is, moreover, to be remarked, with Hewson, that the red corpuscles more readily subside in inflammatory blood from the surface of the whole mass of blood than they will afterwards do from the surface of a mixture with the serum alone.

In an experiment on two portions of a mixture of corpuscles and serum—the one from blood on which no buff formed, the other from blood on which there was a very thick buff—I found that the corpuscles subsided more rapidly in the latter than in the former, though much less rapidly than the separation of the corpuscles from the liquor sanguinis in the formation of the buffy coat takes place.

* Medical Gazette, vol. xxvii. pp. 477-689. Both Mr. Addison and Dr. Barry appear to be unaware that the existence of colourless corpuscles in the buffy coat is no new observation.

† Alison, Outlines of Physiology, Edinburgh, 1839, p. 89.

Hewson's observations led him to remark, that "something more than merely a lessened disposition to coagulate is necessary for the forming of the crust or size." The opinion he entertained was, that the buffy coat probably depended solely upon a change in the coagulable lymph, which, in inflammatory blood, he supposed to be attenuated and specifically lighter; hence, allowing the red corpuscles to subside more rapidly. Dr. John Davy* agrees with Hewson in supposing that the formation of the buffy coat depends chiefly on a greater tenuity of the coagulable lymph. It is to be observed, however, that though blood on which a buffy coat afterwards forms, appears thinner than natural, this is not owing to any increased tenuity of the coagulable lymph, or, more properly speaking, liquor sanguinis, but to a diminution in the number of red corpuscles. Certainly the liquor sanguinis is not less viscid than natural; and, notwithstanding Dr. Davy's inference that there is no necessary connexion between the quantity of fibrin in the blood and its tendency to exhibit the buffy coat, it is now generally admitted that the blood which exhibits the buffy coat in a well-marked manner contains proportionally more fibrin than healthy blood. The opinion of Hewson and Davy thus appears to be untenable, except as regards certain cases in which the blood is unusually thin, from a diminution in the quantity of fibrin, and in which the red corpuscles readily subside, leaving a liquor sanguinis at the top, which yields no consistent buffy coat by coagulation, but merely flakes of fibrin suspended in serum, like moss in water.

In blood in which the buffy coat forms in a well-marked manner there is not only an increase in the quantity of fibrin, but also an increase in the quantity of liquor sanguinis in general, and a diminution in the number of red corpuscles. This relatively greater amount of liquor sanguinis, with its increased proportion of fibrin, is no doubt a condition contributing in some considerable degree to the development of a well-marked buffy coat, but not exactly, as has been supposed, by promoting subsidence of the red corpuscles.

Dr. Alison (*ut supra*, p. 88) is of opinion that the formation of the buffy coat depends on an unusual tendency to separation between the fibrin and corpuscles; but considers it doubtful whether this is owing to increased aggregation among the particles of each, or to a peculiar repulsion between the two. That it is principally owing to an increased aggregation of the red corpuscles, will be shown in the following pages. Here I would observe that any increase in the aggregation of the particles of fibrin would, if it regarded rapidity, lead only to more speedy coagulation; and, if it regarded closeness, to the formation of a more firm coagulum, but not to separation. Besides, before any aggregation at all of the particles of fibrin is manifested, separation of the liquor sanguinis from the red corpuscles has already taken place to the extent of constituting the condition, visible to the naked eye, for the formation of the buffy coat. As to the question of a repulsion between the fibrin, or rather the liquor sanguinis, and red corpuscles, that will be considered below.

The minute process leading to the formation of the buffy coat was, I believe, first explained by Professor Hermann Nasse, of Marburg, and has since been noticed by Professors Rudolph Wagner and Henle. More recently, I have made some observations on the point.

Before entering upon an explanation of the subject it is necessary to call attention to some appearances presented by newly-drawn healthy blood under the microscope. If a drop of such blood spread out by having a thin plate of glass gently laid over it, be quickly transferred to the microscope and forthwith examined, the corpuscles are observed dispersed confusedly about in the liquor sanguinis. In the course of half a minute, however, they are seen to overlap each other, then, rising up on edge, to become fully applied side to side. By this arrangement, like coins in rolls, they occupy less space than when they are irregularly aggregated. The consequence is that the field of the microscope, which was at first uniformly scattered over with corpuscles, now presents spaces containing nothing but liquor sanguinis, with perhaps a single red or colourless corpuscle floating about in it.

* Researches, Physiological and Anatomical, vol. ii. p. 48.—London, 1839.

These spaces represent, as it were, the meshes of an irregular network, formed by the rolls of corpuscles. When the red corpuscles run together into rolls, one may *occasionally* be observed here and there single, but the colourless corpuscles always remain isolated, exhibiting not the slightest attraction for the red corpuscles. After the corpuscles have continued aggregated for a minute or two, a heaving to and fro is usually observed among the rolls, which thus become broken up, and ultimately the corpuscles are more or less detached from each other.

This arrangement of rolls in a *network* is exhibited only when the blood is thinly spread out as above described. When the blood is examined under the microscope in the form of a coagulated drop, the rolls are observed to be disposed in every direction, and thus constitute a *spongework*, in the interstices of which the liquor sanguinis is contained.* A larger quantity of blood, a cupful for example, examined with the naked eye when coagulating, presents on the surface an appearance like jasper; the red mossy-like part being represented by masses of aggregated corpuscles, the transparent instertices by the liquor sanguinis.

What is the nature of the force which causes the mutual approach of the corpuscles and their aggregation into rolls?

A repulsion between the liquor sanguinis and corpuscles, whereby the former tends to separate itself from the latter on a principle similar to the separation of oily fluids from moist substances, has been suggested; but to this it may be replied, that were the force solely of this nature, its effect would be merely to cause an irregular aggregation of the corpuscles, instead of the remarkably regular arrangement which obtains, nor would the corpuscles become detached again from each other, and be promiscuously dispersed through the liquor sanguinis.

The conclusion which a consideration of all the circumstances leads to is, that the force which causes the mutual approach of the corpuscles and their aggregation into rolls, is, in addition to a want of attraction or actual repulsion between the liquor sanguinis and red corpuscles, a special attraction which the latter have for each other, but whether of a vital or physical nature it is not necessary for our purpose to stop here to inquire. It may, however, be remarked that the activity of this attraction is capable of being very much modified by the vital state of the corpuscles on the one hand, and the composition of the fluid in which they are suspended on the other.

The condition on which Dr. Nasse was the first to show that the separation of the liquor sanguinis from the red corpuscles in the formation of the buffy coat principally depends, is an increase in the natural disposition of the red corpuscles to run together.

In that state of the blood in which the buffy coat appears, there is then, together with the known diminution in the quantity of red corpuscles, an exaltation of their natural disposition to run together into rolls, and these again to form a spongework, whereby, the corpuscles being aggregated together more closely, the liquor sanguinis, which is in such cases proportionally increased in quantity, is in a greater measure pressed out as if from a sponge, and of course collects at the top; the corpuscles readily subsiding on account of their greater specific gravity being favoured by closer aggregation.

The view here stated may be illustrated by a sponge soaked in melted tallow—the sponge representing the aggregated corpuscles, the melted tallow the liquor sanguinis. The sponge, let it be supposed, is capable of being drawn together by an intrinsic force, instead of requiring to be pressed together by a force from without.

If, then, before the melted tallow concretes, the sponge be not drawn together, the tallow when concreted will be wholly contained in the meshes of the sponge, though perhaps in greater quantity towards the surface than below. Such is the clot of healthy blood—the spongework formed by the rolls of red corpuscles is not drawn together very closely before the coagulation of the liquor sanguinis takes place,

* This has been pretty well represented in the less magnified of the two figures of a drop of coagulated blood, given by Sir E. Home in the Phil. Trans. for 1818, but the meshes he absurdly describes as being produced by and filled with the carbonic acid gas, which he erroneously supposed was given off by the blood in the act of coagulating.

the coagulated fibrin of this, therefore, is wholly dispersed throughout the meshes, formed by the aggregated corpuscles, but in greater quantity towards the top; hence the greater firmness of the clot there than at the bottom. If on the contrary, before the melted tallow concretes, the sponge be drawn together, a greater or less quantity of the melted tallow will be pressed out and collecting above the contracted, and therefore specifically much heavier sponge, will, when concreted, form a more or less thick cake. Such is an humble illustration of the mode in which the buffy coat is developed. The spongework formed by the rolls of corpuscles is drawn together so closely that the liquor sanguinis is pressed out in more or less considerable quantity, and having collected at the top coagulates, and thus gives rise to the buffy coat. In this case, in consequence of the very great determination of the liquor sanguinis towards the top, the bottom of the mass of blood contains very little of it; hence, after coagulation, the bottom part of the crassamentum is soft and easily broken up from the deficiency of fibrin.

The following cases exemplify the minute process leading to the formation of the buffy coat.

CASE I. Blood drawn from the arm of a young man labouring under pericarditis. By the time a small drop of blood could be transferred to the microscope and observed, the corpuscles had run together into rolls, and these again had become arranged in a netlike manner. The meshes of the network formed by the rolls were very large, and contained nothing but liquor sanguinis. The heaving to and fro of the rolls occurred sooner, and was to a much greater degree than is observed in healthy blood. The disruption of the rolls here and there which ensued was not followed by separation of the corpuscles from each other, but by closer aggregation into isolated masses. The individual corpuscles appeared distinctly thinner than usual, but whether this was connected with their closeness of aggregation—a closeness so great that the corpuscles appeared almost as if fused together—or was owing to a real diminution in thickness, it is impossible to decide, as they were not seen singly in consequence of aggregation into rolls having taken place before any microscopical examination could be made.

The blood drawn from the arm was received into a glass. A thick stratum of liquor sanguinis soon rose to the top, and remained liquid some time. It then coagulated, and formed a thick and well marked buffy coat.

CASE II. The blood drawn from a young man, æt. eighteen, labouring under peritonitis. As the blood was flowing from the vein a minute drop was caught, quickly transferred to the microscope, and immediately examined. The corpuscles had run together into rolls, leaving in the field of the instrument large spaces of liquor sanguinis. The corpuscles appeared thinner and softer than natural, and as if fused together. Heaving to and fro of the rolls, well marked with eventual disruption here and there, and aggregation into heaps. Some of the blood thinly spread out exhibited very distinctly to the naked eye the mottled appearance described by Schroeder Van der Kolk. In the course of some minutes after abstraction, the blood presented a pretty thick buffy coat.

CASE III. The blood of a woman in the last month of pregnancy: aggregation of the corpuscles took place with rapidity, and became very close, leaving large spaces of liquor sanguinis. A buffy coat formed.

CASE IV. Blood drawn from a man with pneumonia of two days' standing. The corpuscles ran together very quickly. They formed here and there very closely aggregated masses of rolls, separated by large intervening spaces. The transverse line of demarcation in the rolls, between the corpuscles, was very indistinct, the corpuscles appearing as if fused together. The corpuscles were more pliable, and appeared as if viscid on the surface. Liquor sanguinis rose to the top of the blood in the vessel, very quickly and in large quantity. After continuing fluid for some time it coagulated, and gave rise to a very thick and firm buffy coat.

But not to multiply cases—let it suffice to say that the numerous observations I have made all agree generally with what has now been stated:—relative diminution in the number of red corpuscles and increase in the quantity of liquor sanguinis;—more rapid and closer aggregation of the red corpuscles into rolls, and these again eventually into masses with large intervening spaces, containing liquor sanguinis;* greater thinness and increased pliability of the red corpuscles, with an appearance frequently of viscidity of their surface, and as if they were fused together: these states have all presented themselves to a degree directly in proportion to the thickness and firmness of the buff which has afterwards formed on the blood, and the extent to which it has become cupped.

Relative diminution in the number of corpuscles and an increased quantity of liquor sanguinis, richer in fibrin, are facts well known regarding inflammatory blood. The more rapid and closer aggregation of the red corpuscles into rolls, and these again eventually into masses with larger intervening spaces, containing liquor sanguinis, now fully ascertained as microscopically characteristic of that state of the blood in which the buffy coat appears, is evidently merely an exaltation of the same attraction which exists in the healthy state. In reference to it, can the increased quantity of fibrin in the liquor sanguinis be looked upon in any other light than as a collateral circumstance? That the fibrin of the liquor sanguinis has no essential influence on the mutual approach of the red corpuscles, and their aggregation into rolls in the healthy state is shown by the fact that the phenomenon takes place in serum after the fibrin has been removed by coagulation or by beating. In buffy blood the corpuscles retain the disposition to run together after the fibrin has been removed longer than in healthy blood. That the increased disposition to aggregate, however, is connected with the increased quantity of fibrin in the liquor sanguinis, would appear from the circumstance that red corpuscles of fresh healthy blood, and even red corpuscles which have had their tendency to run together very much diminished from the length of time the blood has been drawn, run together perhaps somewhat more quickly, when they are mixed with a little of the liquor sanguinis, which has risen to the top to form a buffy coat. To this it may be added, that the red corpuscles, as shown by Nasse, aggregate very rapidly and closely in mucilage of gum, containing a little salt in solution, but this aggregation is irregular and confused in comparison of the regular arrangement of the corpuscles in liquor sanguinis. The material changes observed in the red corpuscles themselves, no doubt, have a direct and intimate connexion with the rapidity and closeness of their aggregation. Besides the changes we have above mentioned, Dr. Nasse says, that the darker the corpuscles are in colour the more quickly do they unite. But the question here rises—what connexion is there between these material changes in the red corpuscles and the material changes in the liquor sanguinis? The appearance of the red corpuscles is known to be very much influenced by the nature of the liquid in which they are suspended, therefore the changes exhibited by the red corpuscles of inflammatory blood may be to some extent owing to the changes in the liquor sanguinis; but, on the other hand, may not the changes in the liquor sanguinis be owing in some degree to the state of the red corpuscles?

It has been observed that the meshes of the network, represented by the peculiar arrangement of the rolls of corpuscles, are larger than are presented by healthy blood under the same circumstances, and that by and by, in consequence of the heaving to and fro, disruption of the rolls takes place here and there, followed by their running together into heaps separated by large intervening spaces of liquor sanguinis. Lest this should lead to the supposition that the spongework arrangement of the rolls of red corpuscles in blood with the buffy coat, must have larger meshes than are found in healthy blood, it is to be remembered, that the large spaces of liquor sanguinis seen under the microscope in a thin stratum of blood are owing to the relative fewness and to the closeness of aggregation of the corpuscles, and that they would in a mass of blood be filled up by other rolls running in all directions, and this very closely, like the fibres of a compressed sponge. The

* In cases in which the blood is thin and watery, large spaces left by the aggregation of the corpuscles may be observed under the microscope, and as mentioned at p. 591, separation of the red corpuscles and liquor sanguinis may take place. This separation, however, appears to be principally the effect of gravity favoured by the little tendency to coagulation, for the changes in the red corpuscles above described are not observed to exist. This circumstance therefore deserves to be well considered in any microscopical examination of the blood, made with a practical view.

result is, as already explained, the meshes of the spongework represented by the peculiar arrangement of the rolls of corpuscles, are in inflammatory blood really very small, and hence contain comparatively little liquor sanguinis. It is however to be remarked that the spongework represented by the aggregated rolls of red corpuscles, in becoming more condensed, is cleft by large fissures, which become filled with liquor sanguinis,—a circumstance tantamount to a sponge being divided into several pieces, but each piece continuing in its compressed state. This, which may be seen on examination before the surface of the blood becomes wholly covered over with the liquor sanguinis, is a higher degree of the state giving rise to the jasper-like appearance above described in healthy blood in the act of coagulating.

The greater size of the spaces between the rolls of red corpuscles in a thin film of blood on which the buffy coat is to form, than in healthy blood, is the cause of the mottled appearance presented to the naked eye, already referred to as having been signalized by Schroeder Van der Kolk, as equally characteristic of the state of the blood as the buffy coat itself. The reason why it is so, it will now be perceived, is, that the appearance is owing to the same cause.

The minute process leading to the separation of the liquor sanguinis from the red corpuscles—the visible condition for the formation of the buffy coat—consists then in an exaltation both of the rapidity and closeness with which the red corpuscles naturally aggregate into rolls, and these again into a spongework, thus squeezing out the liquor sanguinis from among the corpuscles, and allowing the greater specific gravity of the latter to come more fully into play, whereby the liquor sanguinis, which in such cases is in relatively greater quantity, collects at the top, and coagulating, gives rise to the buffy coat.

With a practical knowledge of the appearances above described, it is in our power to infer from the examination of a minute drop of blood drawn from a prick of the finger, as much at least of the state of the blood as can be done from the presence or absence of a buffy coat.

Physiology of the Corpuscles of the Blood. The common way of viewing the blood merely as a fluid has been a great obstacle to the establishment of clear notions regarding its vitality; but if the blood be viewed as a fluid containing suspended in it regularly organized solids, the question of its vitality becomes much more precise, simple and intelligible. The organized corpuscle may as easily be conceived to possess the essential attributes of vitality as any organ in the body; but the liquor sanguinis in which it is suspended is not organized, and can therefore be looked upon merely in the light of a chemical solution,—a solution, however, depending on nicely-balanced affinities kept in play by the vital influence of the corpuscles, and the compositions and decompositions incessantly going on in it. Though not organized and living, the liquor sanguinis, or more properly speaking some of the matters contained in it are strongly disposed to become so under certain conditions. The blood then may be viewed as consisting of organized and living solids, and of a fluid containing in solution matters highly susceptible of organization and life.

The organized and living solids of the blood are the corpuscles, red and colourless.

Nature and Uses of the red Corpuscles. The red corpuscles, according to the best physiologists, are not expended immediately for the purposes of nutrition and secretion. It is from the liquor sanguinis only which permeates the walls of the capillaries, that are derived the materials for nutrition, growth, and the various secretions.

A large number of observations have lately been brought together, however, in defence of the view that the red corpuscles are the material out of which the tissues are directly formed; but Dr. Martin Barry, the author of these observations, has, unfortunately, mistaken changes in the blood-corpuscles, arising from decomposition and from mechanical and chemical agencies for natural vital changes, and has confounded blood-corpuscles with other corpuscles, quite different in their nature. And he has not only failed to demonstrate the link in the chain of evidence required to establish the view he advocates, but has equally failed to adduce any valid arguments against the opposite view.*

If the red corpuscles do not immediately contribute to nutrition, growth, and the secretions, what then is their function, seeing that their presence is as necessary as that of the liquor sanguinis? Do the red corpuscles act merely as "carriers of oxygen,"—or do they in addition to this maintain by their presence the excitability of the organs?

*The red corpuscles considered as carriers of oxygen.* The circumstance that change of tint of the red colour of the corpuscles is the only visible manifestation that the blood has lost or acquired oxygen, has led to the opinion that they are the medium through which that gas is carried to all parts of the system. But there is no reason to suppose that the liquor sanguinis less readily absorbs, and is less a carrier of oxygen than the corpuscles. Moreover it is to be remarked that the absorption of oxygen by the red corpuscles might be looked upon as accessory to some peculiar function performed by them, rather than as being solely for the purpose of distributing the oxygen to the different parts of the system.

Though the red corpuscles may not be mere carriers of oxygen, they still bear a relation to its consumption. Though not themselves expended in nutrition, the red corpuscles are intimately connected with its activity, and that in a manner which it will be endeavoured to explain below. Now as the activity of nutrition has a relation to the amount of oxygen consumed, so also must the activity of the function of the corpuscles.†

* As an example of Dr. Barry's observations, the following may be adduced. He figures and describes muscle in the act of being developed from blood-corpuscles. The subject of his observation was *pressed out with mucus from the fallopian tube of a rabbit* killed ten hours *post coitum.* The red corpuscles, "new cells," were arranging themselves to form muscle. "It is not needful," says he, "to refer to the observations of others, since the objects figured by myself *were obviously muscular fibres* (the future fasciculi) in the earliest stages of formation. There is therefore, it appears, a direct transition of blood discs into the elementary parts of muscle." (Phil. Trans. 1840, Part II, p. 605, Pl. xxx, figs. 14 to 17, inclusive.)

The explanation of the appearance observed, but so grossly misinterpreted, is this:—when blood is mixed with certain muculent secretions, the red corpuscles tend to arrange themselves, as usual, in rolls, but at the same time becoming somewhat distended by the absorption of fluid they appear like rows of beads. Thus if a minute drop of blood drawn from a prick of the finger be mixed with a drop of urethral mucus, and the whole covered with a thin plate of glass and examined under the microscope, the red corpuscles are observed to have become somewhat distended, and to be arranged in many places in single rows, like beads, for the most part parallel. The rows are exactly like what Dr. Barry has delineated, but the most remarkable phenomenon attending this state of the red corpuscles Dr. B. does not appear to have observed,—it is a locomotive power exhibited by the rows of corpuscles. They are observed to move across the field of view somewhat like worms, but very slowly. Even single corpuscles move onwards with a sort of vermicular motion, or like a polygastric infusorium when moving very slowly. This movement appears, however, not to be owing to any contractile power within the corpuscles, but to be determined by attraction for each other and for the aggregations of corpuscles towards which their movements tend. The apparent peristaltic motion appears to be owing to the flaccidity of the corpuscles. A partially filled bladder moving along any surface would present the same appearance. The flacid state of the corpuscles, it is to be remarked, is a necessary condition for its progression, for when a reagent, such as a solution of salt, is applied, the corpuscle shrinks, and is arrested in its movements.

The mistaking of red corpuscles distended by fluid, and arranged in rows like beads, for muscular fibres is, supposing all the attending circumstances of the case abstracted, a conceivable error; but to suppose that blood-corpuscles effused into and mixed with the mucus of the Fallopian tube should there form muscle, is a most extraordinary illusion. For what possible purpose, it may be asked, could muscular fibre be formed in the mucus of the Fallopian tube?

† The relation of the activity of the function of the corpuscles with the amount of oxygen consumed, referred to in the text, it will be seen is indirect, but as in the course of the performance of their function the corpuscles absorb oxygen, and as the oxygen thus absorbed may be accessory to the function of the corpuscles, a direct relation is also to be inferred.

*In addition to being carriers of oxygen do the red corpuscles maintain by their presence the excitability of the organs?* The presence of blood is necessary to maintain the excitability of the organs, but whether the red corpuscles are in this case the sole and direct agents is a question not decided.

John Hunter has remarked that the red corpuscles are connected principally with the strength and vigour of the animal—less with nutrition than with action. But action presupposes nutritive change. The fact appears to be that to "maintain the excitability of the organs," is simply to minister to the nutritive changes which are incessantly going on, and which cannot be stopped without stopping action. Hence, as has been said above, in regard to their relation to the consumption of oxygen, the red corpuscles maintain the excitability of the organs, only inasmuch as they contribute to nutrition. The mode in which they do this, as yet merely alluded to, I now proceed to investigate.

*The red corpuscles considered as glandular cells.* Numerous well-known circumstances combine to show that a process of elaboration goes on within the blood-vessels, whereby matters fitted for assimilation and secretion are prepared from the raw materials entering the blood. As regards the secretions, indeed, some physiologists suppose that they are formed independently in the blood, and are merely separated therefrom by the glands as filters.

The elaboration which goes on within the blood-vessels is partly of a chemical and partly of a vital nature. New chemical compounds are formed, and matters without undergoing any appreciable chemical change are rendered more highly organizable.

What are the agents of this elaboration? While the red corpuscles of the blood have been looked upon as mere carriers of oxygen, or as agents for maintaining the excitability of the organs, the elaboration of the liquor sanguinis out of the various matters poured into the blood-vessels, has generally been attributed to the lungs by those who have justly apprehended some elaboration necessary for the production of the liquor sanguinis—the liquid whence the materials for nutrition and secretion are immediately derived. But the great and perhaps sole function of the lungs is to serve as the medium through which oxygen is taken into, and carbonic acid gas excreted from the blood.

A view has been of late years gaining ground that the special agents of the secretory process are the nucleated corpuscles which constitute the epithelium of the interior of the cells and canals of glands.*

The view just referred to has led to the conjecture that the red corpuscles are the agents of the elaboration of the liquor sanguinis. Wagner remarks that the red corpuscles might be presumed to bear the same relation to the plasma and its normal composition, as the cells of secreting glands do to the secreted fluids;† and in his excellent volume on General Anatomy, Professor Henle, of Zurich, calls the red corpuscles *swimming glandular cells.* It is only necessary to compare for a moment the red corpuscles of the blood with the epithelium corpuscles of glandular surfaces to detect a striking similarity in structure and relations, nor does it require much reflection to perceive the likelihood of an analogy in function.

I agree with Henle in supposing that the red corpuscles draw from the raw materials of the liquor sanguinis a matter, elaborate it, and when elaboration is perfected give back the matter, becoming at the same time melted down in the liquor sanguinis, thus disappearing like the epithelium-cells of glands. In short, as the secretory corpuscles of glands elaborate the secretions from the liquor sanguinis poured out amongst them, so from the new matters constantly entering the blood, the red corpuscles elaborate the liquor sanguinis. The secretory corpuscles of glands are constantly being thrown off, or resolved into a part of the secretion, but are as constantly reproduced; in like manner the red corpuscles of the blood are constantly being melted down into certain of the materials of the liquor sanguinis, but are as constantly being reproduced.

* Purkinje. Report of the Meeting of Naturalists at Prague in 1837. Isis, 1838, No. 7. Dutrochet had well observed that all cells are, properly speaking, secretory organs.

† Physiology, by Dr. Willis. Part II, p. 448.

It is a question how fibrin is formed in the animal body. The true starting-point in nutrition, says Liebig, is albumen. The various protein compounds used as food are, by digestion, all resolved into albumen. In lymph and chyle, some fibrin presents itself, but it is in the blood that that proximate principle is first formed in any considerable quantity, and endowed with the strong and peculiar tendency to become organized. The more peculiar object of the elaboration supposed to be performed by the red corpuscles is probably the conversion of one protein compound into another—albumen into fibrin—a less into a more highly organizable proximate principle.

We have seen that the quantity of fibrin is increased in blood, which shows the buffy coat, and the number of red corpuscles diminished; and we have seen reason to believe from the changes exhibited by the red corpuscles that their action is increased in inflammation, and those other states of the system in which the buffy coat forms on the blood. The result of the increased action of the red corpuscles here assumed, I consider to be the augmentation of fibrin in the liquor sanguinis. This augmentation of fibrin is at the expense, not only of the albumen of the serum but also of the red corpuscles themselves; for by their increased action on the albumen of the serum, the red corpuscles are themselves more quickly exhausted and resolved, therefore, in greater quantity into fibrin than in health.

The Colourless Corpuscles considered in their Relations as one of the Components of the Blood. Leaving out of view the origin and ultimate destination of the colourless corpuscles, it is purposed to consider them here only in their relations as one of the components of the blood. As in blood examined out of the body the colourless corpuscles appear very insignificant, and as an idea of their importance is to be obtained perhaps only by viewing them in the blood as it circulates in the transparent parts of living animals, I would direct attention to the condition of the colourless corpuscles in, and their mode of passage through the minute arteries, the capillaries, and radicles of the veins.

*Condition of the colourless corpuscles in, and their passage through the minute arteries, the capillaries, and radicles of the veins.* When the circulation in the web of the hind-foot of the frog is carefully observed under the microscope, the colourless corpuscles are seen accumulated at the inner surface of the wall of the vessels, along which they move very slowly in comparison of the red corpuscles, which occupy the axis of the current. Besides the difference in rapidity, there is a difference in the mode of progression of the colourless and red corpuscles. Whilst the red corpuscles are carried directly onwards with the liquor sanguinis, the colourless ones roll along over and over like round pebbles at the bottom of a stream of water; sometimes only are they pushed or carried along without rolling. Frequently, when the general current of blood is slow, a number of colourless corpuscles is observed to be stationary, giving to the vessel an appearance as if it were lined with an epithelium of globular corpuscles; a few of which are every now and then becoming detached from the rest and roll along. In the minute arteries when the velocity of the stream of blood is great, the colourless corpuscles are mingled and carried along with the red ones like stones in a rapid current of water; but if the velocity of the stream be diminished, the colourless corpuscles are observed to extricate themselves from among the red ones, and as stones seek the bottom when the force of a current is diminished, come in contact with the wall of the vessel along which they now slowly roll. Through the smaller capillaries the colourless corpuscles pass one by one indiscriminately with the red ones. It is principally in the radicles of the veins that they accumulate in such numbers as actually to line the walls of the vessel like an epithelium.

The peculiar relation of the colourless corpuscles to the walls of the vessels suggested to Poiseuille, (who, it is to be remembered, however, appears not to have perceived any distinction between the colourless and red corpuscles,) the idea that, like what was shown by Girard to take place when a fluid passes through a tube of small diameter, the current of blood is less rapid towards the wall of the vessel, and the stratum in immediate contact with it altogether stationary. This, however, is not altogether a correct view of the phenomenon, as will immediately be seen.

LIBRARY OF THE

Orleans Parish Medical Society

*Attractions and repulsions of the red and colourless corpuscles.* From the facts stated in the preceding part of this paper it may be admitted as fully established that the red corpuscles have an attraction for each other, but none for the colourless corpuscles. The accumulation of the colourless corpuscles at the sides of the vessels proves, as already shown by Ascherson and Weber, the existence of an attraction between them and these walls. The circumstance that the red corpuscles under the ordinary natural circumstances never adhere to the walls of the vessel, is a pretty sure indication of an absence of attraction between these parts, if not of the existence of actual repulsion. The red corpuscles keep together in the axis of the stream by virtue of the attraction they have for each other, but this attraction does not operate within the vessels to so great an extent as is observed in blood just abstracted, otherwise stagnation of the blood would infallibly take place. The cause of this I am inclined to believe is, that when by virtue of attraction contact takes place between the corpuscles, repulsion ensues just as when two bodies which by reason of their being in different states of electricity attract each other, are repelled immediately on contact. The breaking up in the course of a few minutes of the rolls into which the red corpuscles aggregate immediately when drawn, above described, is owing, perhaps, to an imperfect exertion of the same repulsion, or at least cessation of attraction between the red corpuscles here supposed to supervene on contact.

A knowledge of the attractions and repulsions just mentioned appears calculated to throw some light on the circulation in the capillaries including the terminations of the arteries and radicles of the veins.

*Circulation of the blood in the capillaries, including the terminations of the arteries and radicles of the veins.* From the circumstance that the liquor sanguinis passes through the walls of the minute vessels by imbibition, it is to be inferred that there is an attraction between it and these walls. This being the case, the liquor sanguinis of the circulating blood in contact with the walls of the vessels will be retarded in its course, just as takes place in the passage of water through narrow tubes of glass. The red corpuscles, by virtue of their attraction for each other and repulsion, or want of attraction, for the walls of the vessels, keep in the axis of the stream, whilst the colourless corpuscles, by virtue of their attraction for the walls of the vessels, and their want of attraction, or repulsion, for the red corpuscles, apply themselves to the walls. Being thus in the less rapid stratum of liquor sanguinis they are either not at all or very slowly carried along. The rolling over and over mode of progression which they so often exhibit, appears to me to be caused by the onward movement of the string of red corpuscles aggregated in the axis of the stream, acting in the same way as a log of wood does in carrying along with it the balls or rollers placed underneath, in order that it may be moved more easily. It is indeed probable that the colourless corpuscles do actually in this way facilitate, or at least offer less obstruction to the course of the stream of red corpuscles than if, considering their attraction for the walls of the vessels, they had to have been pushed along. It is scarcely necessary to remark that the mode of progression of the colourless corpuscles under consideration explains the slowness of their course in comparison of that of the stream of red corpuscles.

It has been stated that in the minute arteries, when the velocity of the stream of blood is great, the colourless are mingled and carried along with the red corpuscles. In this case the attraction between the colourless corpuscles and walls of the vessels is overcome by the force of the current, in the same way as rapid waters overcome the force of gravitation, by raising up from the bottom, suspending, and carrying along even very large stones. As when the force of the stream of water subsides, the stones by virtue of the attraction of gravitation again seek the bottom, so when the force of the stream of blood is diminished by any cause, the colourless corpuscles, by reason of their want of attraction for the red ones, are extricated from among them, and by virtue of their attraction for the wall of the vessel are brought into contact with it.

*Mode in which arrrestment of the circulation takes place in the capillaries.* The absence of attraction or the existence of actual repulsion between the red corpuscles on the one hand, and the walls of the vessels and colourless corpuscles on the other, is a most important fact to keep in view. Without this absence of attraction or existence of actual repulsion the passage of the blood through the small vessels would have been impossible. Indeed it is a change in the attractions and repulsions among the red corpuscles which appears to be the cause of inflammatory congestion.

When any irritating substance, a solution of common salt for example, is applied to the web of the frog's hind-foot, or when the part is wounded, the congestion which supervenes on the temporarily accelerated circulation, is observed under the microscope to commence by the red corpuscles agglomerating together and applying themselves here and there flat against the wall of the vessel, and adhering to it.* Other red corpuscles apply themselves to those already adherent and complete stagnation ensues. The blood in the lungs of the frog is observed to be arrested in the same way in the vessels when the part of the lung under observation is touched with solution of salt, or, as I have also found on making the experiment, *when a stream of carbonic acid gas is directed against it.*

The stoppage of the circulation in the capillaries which occurred in Mr. Blake's experiments of injecting different salts into the blood is to be attributed to the same change in the attractions and repulsions of the red corpuscles which is here considered as the immediate cause of the stoppage of the capillary circulation in the cases above described. The stoppage of the circulation in the capillaries of the lungs in asphyxia, it may be inferred from what is above stated of the action of carbonic acid gas, is owing to the same cause.

*The colourless contrasted with the red corpuscles in their relations to the nutritive process.* In contemplating in their relations with nutrition, &c., the phenomena just described, the first thing that strikes us is the *distended* and *globular* colourless corpuscles, rolling *slowly* along the walls of the minute vessels, whilst the *collapsed flattened* red corpuscles proceed *rapidly* onwards in the axis of the stream. The very natural inference from this is that, as Weber has already observed, there is some reciprocal relation between the colourless corpuscles, and the parts outside the vessels in the process of nutrition; whilst, as I have above endeavoured to show, the red corpuscles have no direct relation with the parts outside the vessels, but are more concerned in the elaboration of the liquor sanguinis.

*Cause of the variations in the capillary circulation.* It is interesting to consider the different capabilities for endosmose and exosmose, in reference to the liquor sanguinis, possessed by the red and colourless corpuscles, as indicated by their different states of distention, and to compare this with the difference in the attractions and repulsions they exhibit. The changes constantly going on in the blood is attended with variations in the capabilities of the corpuscles for endosmose, and in their attractions and repulsions. These appear to be the cause of the variations which are constantly occurring in the capillary circulation. The force of the heart alone, and not any action of the capillaries, determines the general passage of the blood from the arteries into the veins, but it is to the attractions and repulsions of the corpuscles that the varied peculiar movements of the blood in the capillaries are owing. In considering the circulation, through the capillaries, in short, it is always to be remembered that the blood is not a mere inert fluid, but one containing, in suspension, innumerable organized and living corpuscles endowed with peculiar attractions and repulsions.

The view now given of the nature and uses of the corpuscles of the blood appears calculated to guide to a more correct explanation of many obscure points in physiology and pathology. Without necessitating us to give up any of the arguments of solidism, it puts into our hands all the valuable ones of humorism.

* The observation of Weber that red corpuscles sometimes adhere to the walls of the vessels and are changed into colourless ones, has not been confirmed. All my observations are against it.

